# thebeat: A Python package for working with rhythms and other temporal sequences

**DOI:** 10.3758/s13428-023-02334-8

**Published:** 2024-02-02

**Authors:** J. van der Werff, Andrea Ravignani, Yannick Jadoul

**Affiliations:** 1 Comparative Bioacoustics Group, Max Planck Institute for Psycholinguistics, Wundtlaan 1, Nijmegen, The Netherlands; 2 Department of Human Neurosciences, Sapienza University of Rome, Piazzale Aldo Moro, 5, Rome, Italy; 3 Center for Music in the Brain, Aarhus University, Universitetsbyen 3, Aarhus, Denmark

**Keywords:** Python, Music, Rhythm, Timing, Acoustics, Bioacoustics

## Abstract

*thebeat* is a Python package for working with temporal sequences and rhythms in the behavioral and cognitive sciences, as well as in bioacoustics. It provides functionality for creating experimental stimuli, and for visualizing and analyzing temporal data. Sequences, sounds, and experimental trials can be generated using single lines of code. *thebeat* contains functions for calculating common rhythmic measures, such as interval ratios, and for producing plots, such as circular histograms. *thebeat* saves researchers time when creating experiments, and provides the first steps in collecting widely accepted methods for use in timing research. *thebeat* is an open-source, on-going, and collaborative project, and can be extended for use in specialized subfields. *thebeat* integrates easily with the existing Python ecosystem, allowing one to combine our tested code with custom-made scripts. The package was specifically designed to be useful for both skilled and novice programmers. *thebeat* provides a foundation for working with temporal sequences onto which additional functionality can be built. This combination of specificity and plasticity should facilitate research in multiple research contexts and fields of study.

## Introduction

**Fig. 1:**
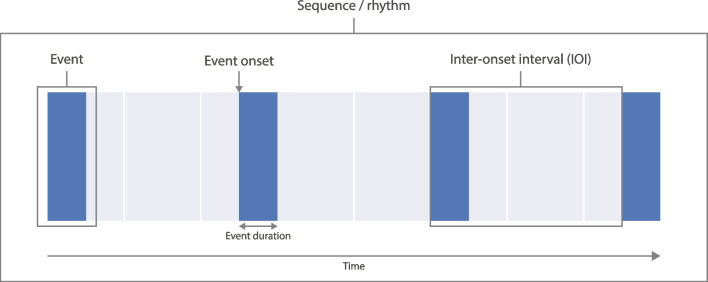
Example of a temporal sequence or rhythm. Different types of temporal sequences share a similar underlying structure. Events are placed at different points on the time *x* axis. The event onsets are the exact *t* values at which the events occur, and inter-onset intervals (IOIs) are the intervals between the onset of one event and the onset of the next event. In addition, each event has a duration

Research involving temporal sequences (e.g., rhythms) often relies on similar experimental principles. Events, such as sounds, are placed at different points in time, possibly with empty intervals between them (Fig. 1). Together, the events may form a sequence, a rhythm, or a click train. The analysis of temporal sequences involves universally applicable methods, such as calculating inter-onset intervals (IOIs) or autocorrelations (Ravignani & Norton, 2017). Temporal sequences are therefore created and analyzed using largely equivalent computer scripts. For example: different MATLAB scripts were written for creating sound stimuli in two experiments that differed only in the used parameters (Celma-Miralles & Toro, 2020; Zeni & Holmes, 2018). To date, no commonly available software package exists that can prevent such repetition, which is why we present *thebeat*. The package is completely focused on creating, analyzing, and visualizing temporal data. *thebeat* is available from https://github.com/jellevanderwerff/thebeat.

**Table 1:** Example uses of *thebeat* and example studies or reference papers

Stimulus generation	
Auditory stream segregation	Simon and Winkler (2018)
Click trains	Roach et al. (2019)
Fast periodic auditory stimulation	Barbero et al. (2021)
Finger-tapping	Guérin et al. (2021)
Isochrony	Horr and Di Luca (2014)
Psycholinguistics	Bosker (2017)
Rhythm perception/production	Repp et al. (2005)
Rhythmic auditory stimulation (RAS)	Gonzalez-Hoelling et al. (2022)
Rhythmic grouping	Iversen et al. (2008)
Sensorimotor synchronization	Merchant et al. (2005)
Speech segmentation	Thornton et al. (2018)
Temporal regularity	Celma-Miralles and Toro (2020)
Tone clouds	Bianco et al. (2020)
Data visualization	
Circular histograms	Kirschner and Tomasello (2009)
Melodies in musical notation	Morgan et al. (2019)
Recurrence plots	Burchardt et al. (2021)
Phase space plots	Ravignani (2017)
Rhythms in musical notation	Repp et al. (2005)
Sequences as event plots	Bouwer et al. (2016)
Data analysis	
Autocorrelations, cross-correlations	Ravignani and Norton (2017)
Beat-finding (Fourier, autocorrelations, etc.)	Burchardt et al. (2021)
Coefficient of variation, ugof	Burchardt et al. (2021)
nPVI	Patel and Daniele (2003)
Shannon entropy, grammatical complexity	Lumaca and Baggio (2020)

*thebeat* helps to reduce the time researchers spend programming custom computer scripts when working with temporal sequences. We see two additional advantages for its use: First, inexperienced programmers will benefit from our extensively tested code when creating experiments or analyzing data. Warnings are issued in situations that can lead to commonly made errors. Second, *thebeat* facilitates the replication of studies, currently hindered by the use of idiosyncratic computer scripts. We hope that *thebeat* will provide consistency in how we create, analyze, and report about temporal sequences and rhythms, providing a straightforward environment in which to create and explore temporal data. *thebeat* was designed so as to not require any in-depth knowledge of computational concepts such as signal processing or array programming, and can thus be used in educational settings.

Table 1 provides examples of contexts in which *thebeat* will help speed up the research process, and where it can make the methods that are used more comparable. At present, most of *thebeat*’s functionality is focused on sound, but fundamentally it is not tied to any perceptual domain. *thebeat*’s modules are organized around three parts of the research process: stimulus generation, data visualization, and data analysis. We here describe a few examples of functionality that is included in the package. For a similar overview, but with direct links to *thebeat*’s functions, the reader is referred to the package documentation (https://thebeat.readthedocs.io). *thebeat* contains a variety of functions for creating and manipulating sequences or rhythms based on event onsets, IOIs, note values, and interval ratios. Importantly, it allows easy conversion between all these types of timing data. *thebeat* also offers functions for generating random timing data and random rhythms. For creating stimuli, *thebeat* includes functions for synthesizing sounds, but it can also use existing sound files or import *Praat* sound objects through *Parselmouth* (Boersma & Weenink, 2022; Jadoul et al., 2018, 2023). For visualizing temporal data, *thebeat* can be used to create waveforms, event plots, and musical notation, as well as more complex plots, such as recurrence plots (Burchardt, Picciulin, Parmentier, & Bolgan, 2021), phase space plots (Ravignani, 2017), and rose plots (circular histograms; Ravignani and Norton, 2017). For the analysis of temporal data, *thebeat* can calculate cross-correlations and phase differences (cf. Ravignani and Norton, 2017), and can perform beat extraction using autocorrelations or Fourier transforms (Ravignani & Norton, 2017). As a final example, rhythmic complexity can be calculated using measures such as information entropy and edit distance (Lumaca & Baggio, 2017; Ravignani & Norton, 2017).

*thebeat* was designed with users of varying programming proficiency in mind. The core focus was not on providing a large amount of functionality, but rather on providing simple methods with reliable internal checks and verifications. To allow for exploratory programming, we believe it is important that users obtain results in as few lines of code as possible, without compromising on code intelligibility and flexibility. In fact, most functions achieve the desired result in one line of code. Functionality was only implemented if useful in research contexts and if not better handled by a more specialized package. As an example, simple sounds can be created using *thebeat* (useful for most research contexts), but advanced audio manipulation is better performed using packages such as *Parselmouth* (Jadoul et al., 2018) or *librosa* (McFee et al., 2015).

*thebeat* is an on-going, open-source, and collaborative project distributed under the GPL-3 license. We encourage its users to contribute and make suggestions, or to start discussions about the idiom and methods used. To that end, we provide a GitHub repository (https://github.com/jellevanderwerff/thebeat) and a Gitter chatroom (https://gitter.im/jellevanderwerff/thebeat. Extensive documentation is available from https://thebeat.readthedocs.io. The documentation includes detailed descriptions of package functionality and many examples that show what the package can do. In addition, it contains tutorial-style examples in which methods sections from existing research are replicated. We hope to have created a strong foundation onto which additional functionality can be built, and we encourage *thebeat*’s users to request or contribute functionality useful for timing and rhythm research.

## Package principles

**Fig. 2:**
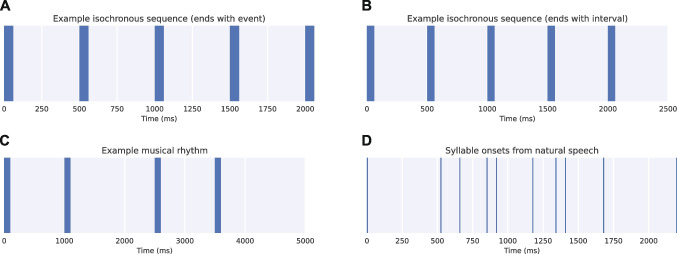
Examples of different sequences plotted using *thebeat*. For panels **A**–**C**, the thickness of the *blue lines* represents the events’ durations. For panel *D*, the *blue lines* represent the boundaries between the inter-onset intervals, rather than the durations of the events. Panels **A**–**C** are examples of SoundSequence objects (which also contain information about the events), whereas panel **D** is an example of a Sequence object, which always contains only timing information but no information about the events themselves. **(A)** A sequence that ends with an event, based on Yee et al. (1994). **(B)** A sequence that ends with an interval, based on Yee et al. (1994). **(C)** Event plot for a musical rhythm, based on Repp et al. (2005). **(D)** Event plot for a spoken Dutch sentence, based on van Son et al. (2001)

Figure 2 shows examples of different types of temporal sequences and rhythms. For describing the temporal structure of sequences, *thebeat* encourages using IOIs, for three reasons. First, IOIs are onset-to-onset—and not offset-to-onset—and so can be universally used for different types of sequences, whether they be musical rhythms or click trains. Second, IOIs induce the beat or rhythm percept, at least in human listeners (Parncutt, 1994). Third, the structure of IOIs is preserved when the durations of the events change.

In *thebeat*, two types of sequences are distinguished: sequences that end with an event (Fig. 2A), and sequences that end with an interval (Fig. 2B and C). Sequences that end with an event contain *n* events, but $n-1$ IOIs. Sequences that end with an interval contain an equal number of events and IOIs. *thebeat*’s default option is for sequences to end with an event. Rhythms, or two sequences that need to be concatenated, are required to end with an interval. This is necessary because otherwise the last event of a sequence and the first event of a following sequence would coincide.

**Fig. 3:**
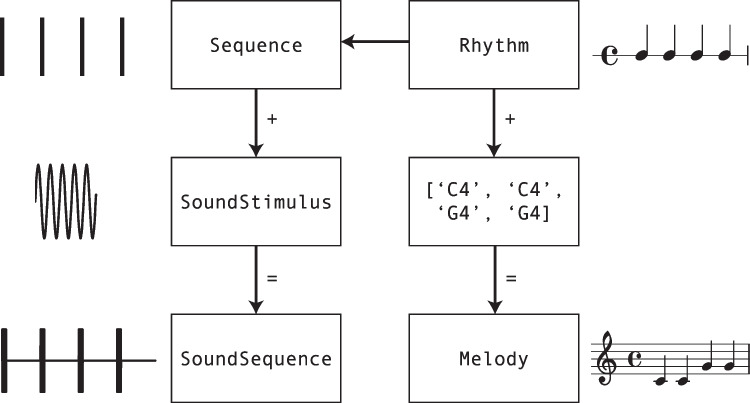
The different object classes used in *thebeat*. Sequence objects contain timing information, SoundStimulus objects contain a stimulus sound. Together they combine into a SoundSequence object, representing a trial. Rhythm objects can either be converted to a Sequence object, or combined with a list of pitch names into a Melody object

*thebeat*’s core functionality revolves around three major concepts: sequences, sound stimuli, and sound sequences. These are implemented as different Python classes (respectively, Sequence, SoundStimulus, and SoundSequence). For musical contexts, two additional concepts exist: rhythms (the Rhythm class) and melodies (the Melody class). In Python, classes are simply variable types that contain specific functions (methods). Figure 3 shows how to combine and convert between classes.

The most important class is the Sequence. It contains timing information, but no information about the events themselves, such as their duration. Figure 2D is an example of a Sequence: it contains only information about the intervals between the onsets of the events (here, the onsets of the syllables) but no information about the produced sounds. As such, the plotted lines are of arbitrary width, and only indicate the intervals’ boundaries. A Sequence object is agnostic about the used time unit (seconds, milliseconds, etc.), and so can flexibly integrate with different datasets and measurements, and other programs and packages. Together with a SoundStimulus object—which represents the audio of a single stimulus—Sequence objects combine into a SoundSequence object, which holds timing information as well as audio. Figure 2A–C are examples of SoundSequence objects. One SoundSequence object in rhythm or timing research is equivalent to one trial. A Rhythm is a special kind of Sequence: in addition to containing timing information, it has a time signature, a value for the duration of a single beat in milliseconds (based on the denominator of the time signature), and an indication of which events to play (to allow for musical rests). Finally, Melody objects are a combination of a Rhythm object and a list of pitch names. For all these classes, plotting functions are included (producing event plots, waveforms, or musical notation). SoundStimulus, SoundSequence, and Melody objects can additionally be played, or written to disk as a sound file.

## Getting started with *thebeat*

### Installation and dependencies

*thebeat* can be installed from the Python package repository PyPI by typing pip install thebeat into a command window or terminal. This installs a basic version of *thebeat*. To additionally install *thebeat*’s functionality for plotting musical notation, use pip install ’thebeat[music_notation]’. *thebeat* is consistently tested on Windows, Linux, and Mac operating systems. During installation, the main dependencies necessary for most of the functionality are automatically installed: *NumPy* (Harris et al., 2020), *SciPy* (Virtanen et al., 2020), *pandas* (McKinney, 2010), and *Matplotlib* (Hunter, 2007). If installing with the functionality to plot musical notation, the packages *abjad* (Bača et al., 2015) and *lilypond* (Nienhuys & Nieuwenhuizen, 2003) are additionally installed. More information can be found in the *Installation* section of the package documentation.

### Creating a simple trial

Below we use *thebeat* to create a simple isochronous (i.e., regular) trial of ten events at a tempo of 500 ms (i.e., each IOI is 500 ms, corresponding to 120 bpm or 2 Hz). It contains only pure tones of 50 ms with a pitch of 440 Hz.
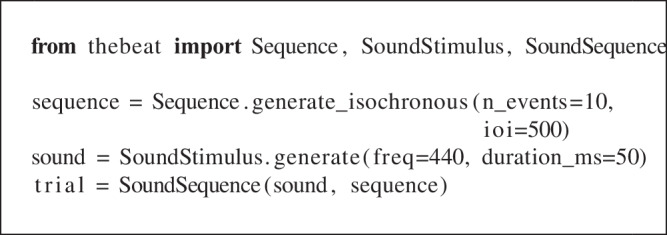


We start by importing the required object classes from *thebeat* and by creating a Sequence object using its Sequence.generate_isochronous() method. As mentioned, by default a Sequence object ends with an event, and so seq contains ten events, but nine IOIs. We then use the SoundStimulus.generate() method to synthesize a single sound stimulus. Finally, we combine the Sequence and SoundStimulus objects into a SoundSequence object, which can be played back, plotted, or written to disk (respectively, trial.play(), trial.plot_waveform(), or trial.write_wav()).

Below we illustrate how *thebeat* may be used in practice using three code examples. In the first, we create a regular sound sequence and a randomly timed sound sequence. Sequences like these are common in auditory timing research. In the second example, we create and plot rhythms and melodies. In the final example, we demonstrate how *thebeat* can be used for analyzing empirical temporal data using a dataset of sperm whale vocalizations. More examples can be found in the package documentation (https://thebeat.readthedocs.io).

## Example 1: Creating stimuli for an auditory perception experiment

Here we use *thebeat* to create two types of trials: a regular trial and a randomly timed trial for which we sample the IOIs from a uniform distribution. Both trials contain ten events. For the regular trial we combine two isochronous sequences of five events and place a random inter-trial interval (ITI) between them. The regular trial contains ten identical pure tones, and the randomly timed trial contains ten identical complex sounds. We start by creating the Sequence objects, which always contain only timing information:
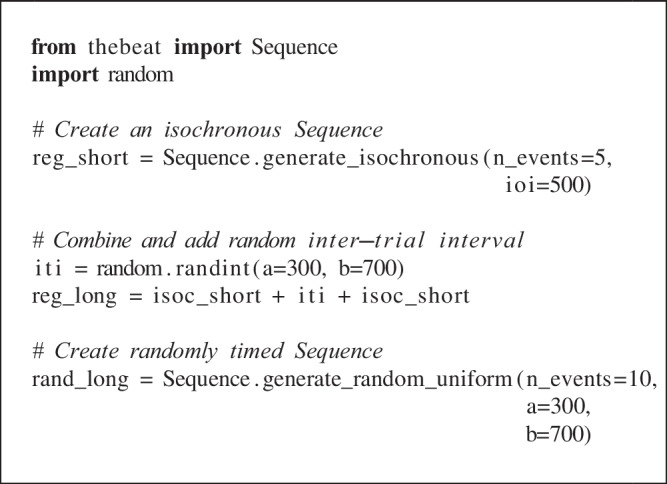


This code demonstrates a few basic functionalities of *thebeat*. Sequences can be created or randomly generated in a variety of ways, and the resulting Sequence objects can then be manipulated using standard operators. The plus-operator can be used for joining different sequences, or a sequence and a number representing an interval, together. Similarly, the multiplication operator might be used for repeating sequences.

Next, we create two different sounds: a pure tone sound, and a complex sound composed of a sine wave and its first two harmonics:
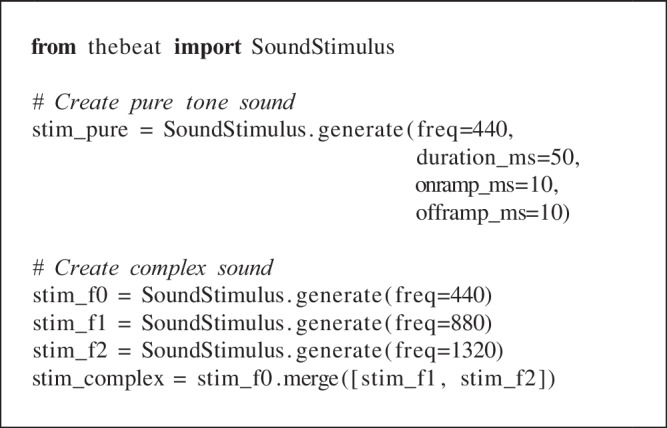


On- and offramps (i.e., attack and decay) can be specified using the onramp_ms and offramp_ms arguments. Complex sounds can be created by combining existing SoundStimulus objects using their .merge() method.

Finally, the code below shows how to combine the Sequence object’s timing information with the SoundStimulus object’s acoustic information into a trial (a SoundSequence object). We can pass SoundSe-quence either a single SoundStimulus object, in which case the same sound is used throughout, or a list of SoundStimulus objects, in which case a different sound is used for each respective event. Here, for the isochronous trial we use the created pure tone sound. For the random trial, we use the created complex sound:
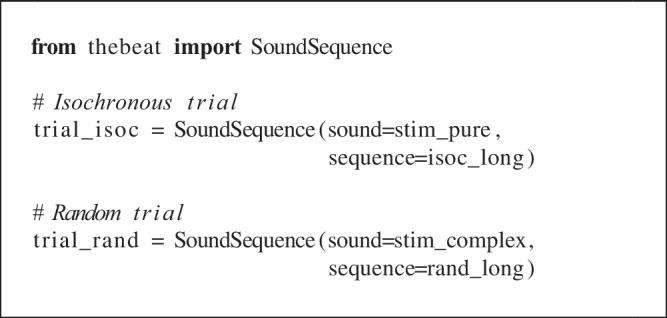


These trials are ready to be used in an experiment after saving them to a .wav file, e.g., trial_isoc.write_wav(‘file.wav’). Trials can also be created on the fly without saving for use with other Python packages, such as PsychoPy (Peirce et al., 2019).

## Example 2: Rhythms and melodies

### Rhythms

Python is not a specialized music-making program, and so for creating rhythms and melodies researchers will often resort to commercial programs such as Ableton, Max, or GarageBand. *thebeat* offers enough functionality for working with rhythms and melodies as required by most experimental methodologies. We can create them using simple commands, synthesize them into sound, or plot them in musical notation. We can also generate random rhythms and melodies after specifying a few constraints.

**Fig. 4:**
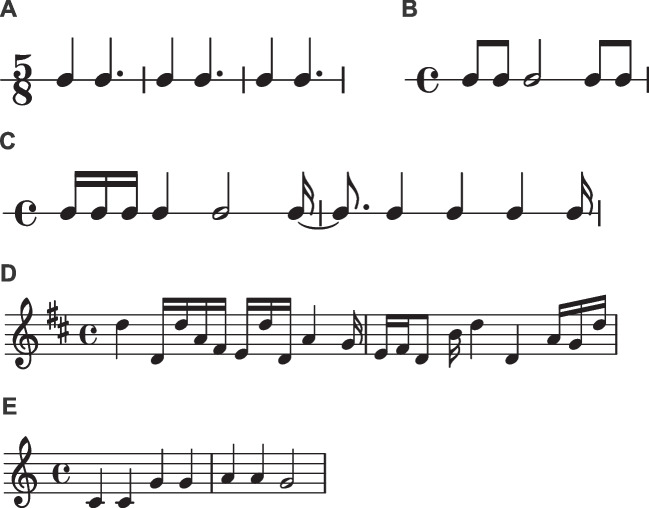
Example rhythms and melodies, plotted using *thebeat*

Rhythm objects can be created from integer ratios or note values, or from IOIs. The first, simple rhythm in the example was used in Repp et al. (2005). We create it using integer ratios. To illustrate, the integer ratios [1, 1, 2] describe a sequence where the final note is twice as long as the first two notes (e.g., Jacoby and McDermott, 2017). Different from Sequence objects, Rhythm objects require a time signature and a duration for the beat in milliseconds. Here, ‘beat’ refers to the denominator of the time signature. In 5/8, the beat duration thus represents the duration of an eighth note, whereas in 5/4 it represents the duration of a quarter note. We plot the rhythm in musical notation using the Rhythm.plot_rhythm() method (Fig. 4A).

For some research contexts, it is useful to be able to produce random rhythms given a few constraints. The second rhythm in the example is created using the Rhythm.generate_random_rhythm() method. This method chooses, with equal probability, a rhythm out of all possible rhythms given some desired constraints. As constraints, we pass it the desired number of musical bars, the note values that may be used, and the duration of one beat. The rhythm created in the example contains one bar filled with half notes, quarter notes, or eighth notes. It has a time signature of 4/4 and a beat duration of 500 ms.
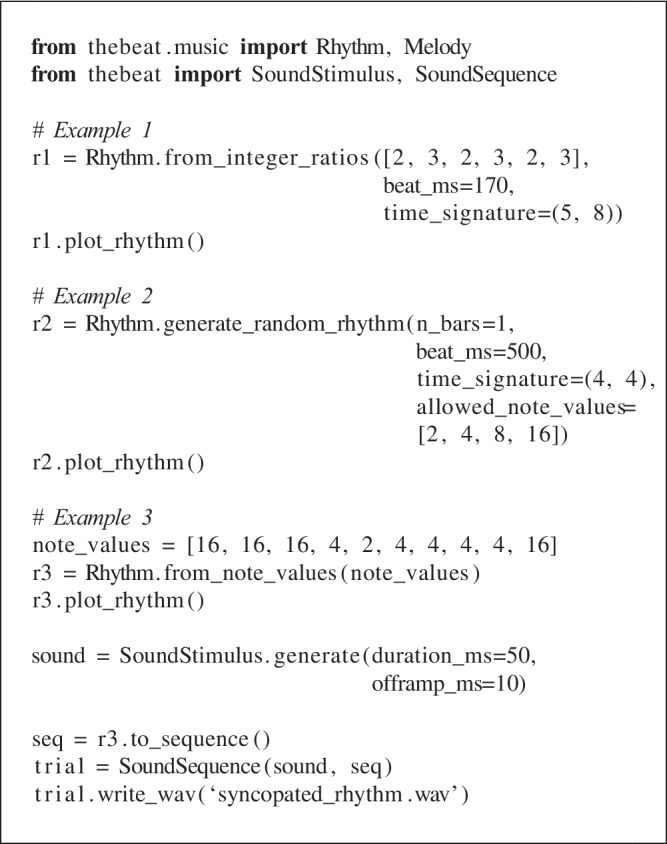


The final example shows how we might create a syncopated rhythm (Fig. 4C). In the example, we create it from a list of note values, where e.g. 4 refers to the denominator of the 1/4 note value. We then plot the Rhythm object, convert it to a Sequence object, add sound, and save the file to disk. The resulting .wav file can be found in the Supplementary Materials.

### Melodies

*thebeat* contains basic functionality for creating melodies. In the code example, we create a random melody (Fig. 4D) and a melody that we create using note names (Fig. 4E).

In the first example, the Melody.generate_ran-dom_melody() method is used for creating a random melody in the key of D (Fig. 4D). Random melodies can be generated for different keys and octaves, though note that this method does not impose any harmonic ‘rules’ (such as starting or ending on the tonic). In the second example, we manually create a melody by combining a Rhythm object with a list of pitch names. Pitch names can be input as, for instance, ‘G’, or as ‘G4’, where the number refers to the used octave. After the Melody object has been created, we can plot it (Fig. 4E), or synthesize it into sound and save the sound to disk. By default, when synthesizing a melody into sound, the sounds have durations that are equal to the provided note values. In the example, this would mean that each quarter note lasts 500 ms. In most cases this is undesirable, and so we require all sounds to have a duration of 50 ms. Note that the melody’s rhythmic structure is preserved. The resulting file again can be found in the Supplementary Materials.
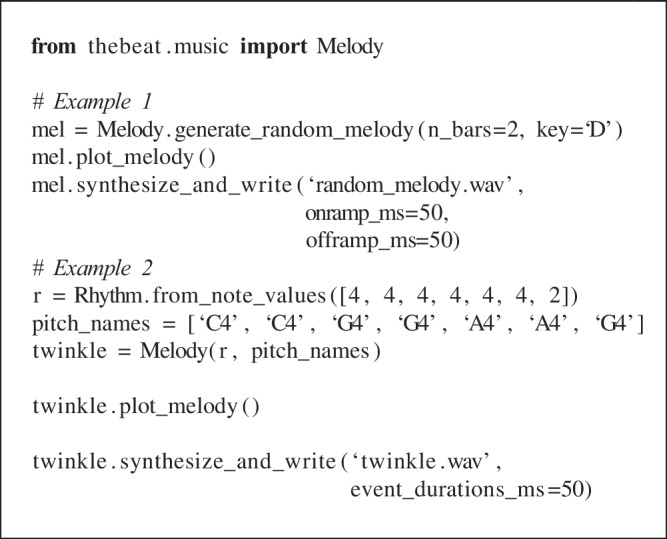


## Example 3: Analyzing sperm whale codas

**Fig. 5:**
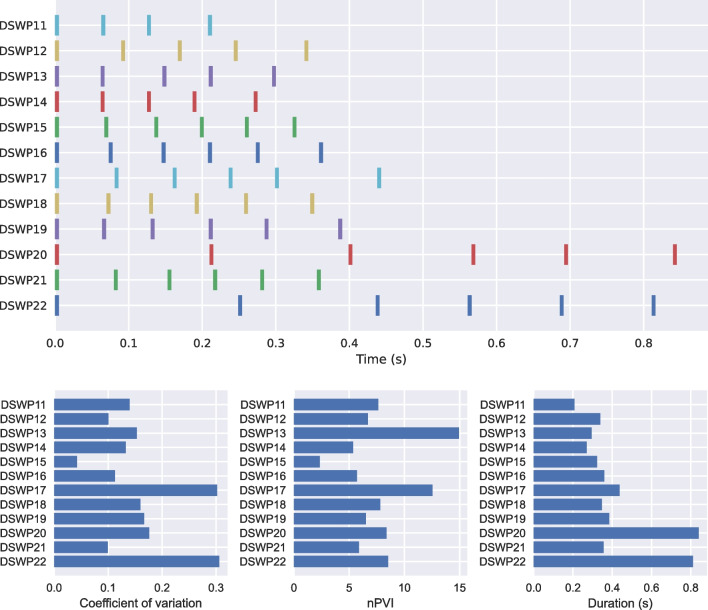
The first 12 sperm whale codas from the 2008 recordings from the dataset in Hersh et al. (2021). *Top*: event plot showing the codas as a function of time. *Bottom*: the coefficient of variation, nPVI, and Shannon entropy for the codas. The code for plotting the bottom row of barplots is provided in the Supplementary Materials

**Fig. 6:**
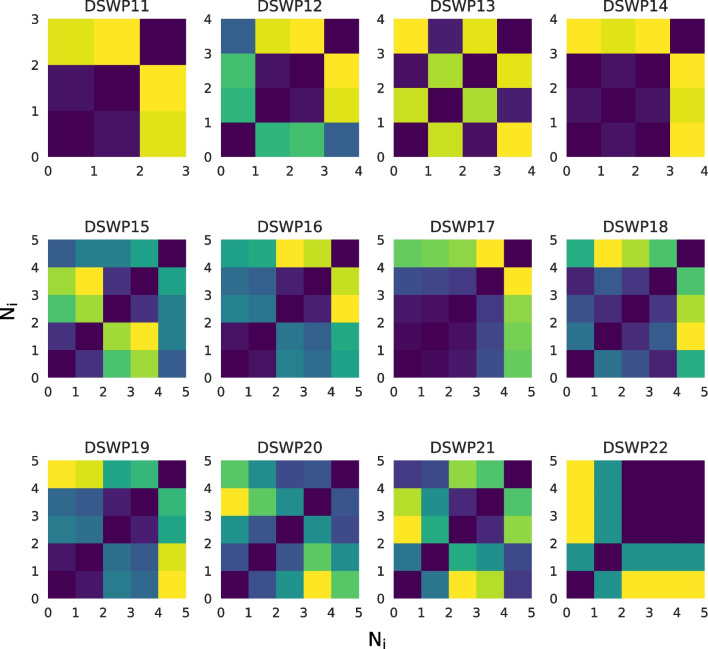
Recurrence plots for the first 12 codas in the sperm whale dataset from Hersh et al. (2021)

Sperm whales live in so-called clans that have signature communicative signals by which they can be identified (Hersh, Gero, Rendell, & Whitehead, 2021). These vocalizations, or ‘codas’, consist of a pattern of ‘clicks’. Here, we use two of *thebeat*’s plotting functions to visually contrast types of sperm whale codas (Figs. 5 and 6), and calculate commonly used statistics for describing them (Fig. 5). We use an abridged version of the dataset from Hersh et al. (2021) that contains only recordings from 2008. The dataset can be found in the Supplementary Materials. Since these data are already based on IOIs, we can create the objects by simply passing the IOIs to the Sequence constructor (see code example).

We start by importing the necessary packages, and loading the sperm whale data using *pandas* (McKinney, 2010). We create a Sequence object for each of the distinct calls—in the dataset uniquely identified by codanum—and pass the codanum to the name argument. We then save the objects to a list. For identifying different types of codas we plot them in an event plot. We use *thebeat*’s plot_multiple_sequences() to do so. We pass arbitrary linewidths because these data do not contain information about the duration of the clicks. Contrary to the previous examples, the IOIs are in seconds instead of milliseconds. Different rhythmic patterns in the codas can now be visually identified (Fig. 5).

To visualize these patterns in more detail, we can use one of *thebeat*’s other plotting functions. Here, we create recurrence plots (Ravignani & Norton, 2017). The patterns that emerge in these plots can be used for comparing sequences’ underlying rhythmic structures (Burchardt et al., 2021). In the code example, we plot recurrence plots for the first 12 sperm whale codas.

After creating a grid of 12 *Matplotlib* Axes objects (i.e., subplots), the recurrence plots can be produced using the recurrence_plot function from *thebeat*’s visualization module. All plotting functions in *thebeat* allow plotting onto an existing *Matplotlib* Axes object by passing the Axes object to the function’s ax argument. Looping over the codas, we can thus create a recurrence plot for each of the 12 subplots. Among other things, the resulting plot (Fig. 6) reveals that, even though coda *DSWP11* has fewer events than *DSWP14*, the two codas have a similar underlying structure.
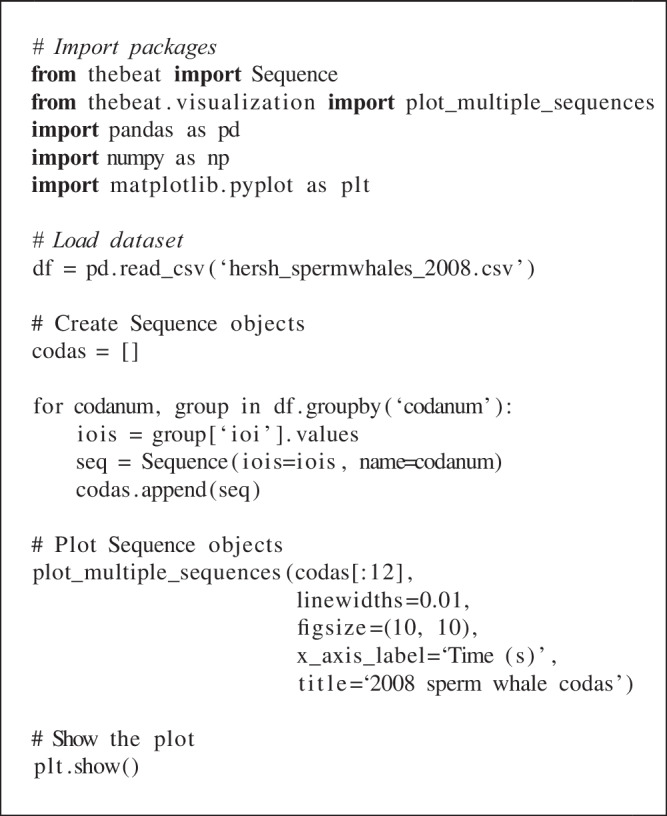

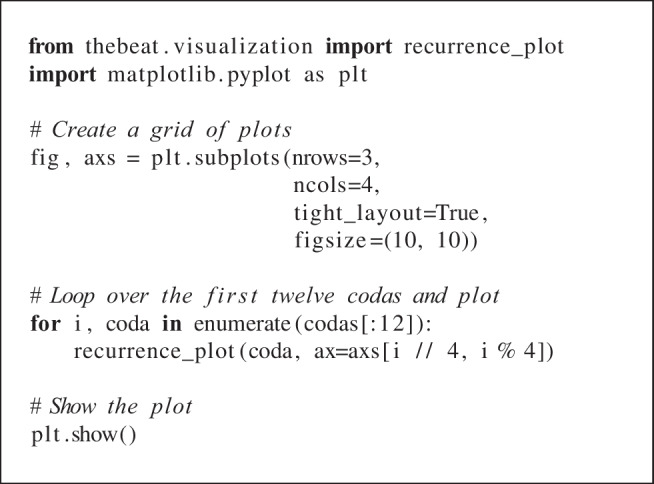


Finally, we use *thebeat*’s statistics module to calculate the normalized Pairwise Variability Index (nPVI; Patel and Daniele, 2003), the coefficient of variation (cf. Burchardt et al., 2021), and Shannon entropy (Shannon, 1948). These statistics measure how much variability there is between the IOIs in each of the codas (Fig. 5):
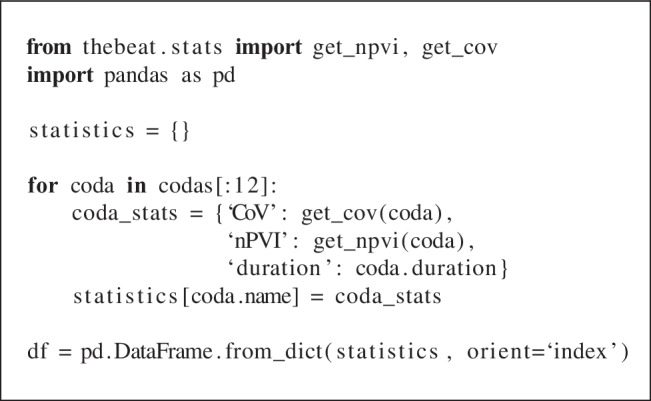

## Discussion

The examples above illustrate situations where researchers can benefit from using *thebeat*. They also show that *thebeat* can be combined with existing packages, such as *NumPy* and *Matplotlib*. We created *thebeat* to standardize how we work with temporal sequences and rhythms in the behavioral and cognitive sciences, and we believe that its use will advance research in five main ways. First, *thebeat* provides consistent and quick methods for those aspects of temporal research that are universal across methodologies. Rather than having to write scripts to convert between event onsets, IOIs, or integer ratios, these operations can now be performed using single lines of code. Second, temporal data analysis is often complicated by the use of different in- and output formats. *thebeat*’s Sequence class provides a central data type that can be used as the start- and endpoint for different analysis methods. Third, ease of use makes *thebeat* accessible to researchers new to the field, allowing easy visualization and analysis of temporal data. Fourth, *thebeat* is extensively tested, and issues warnings in cases where its users might expect a different output than the output produced. It also provides helpful error messages in cases where operations are attempted that are logically (or mathematically) impossible. Finally, the package documentation includes detailed descriptions and explanations for each method, as well as references to relevant literature.

While *thebeat* contains functionality for analyzing and visualizing temporal data, it does not contain functionality for automated analysis of audio or video. The input is expected to either be pre-processed event onsets or IOIs. Even though it was a deliberate choice to not include any methods for raw data analysis, this might be reconsidered in the future. Moreover, *thebeat* for now can only handle sequences and rhythms that contain a single stream of events. In the future we may integrate functionality for working with complex sequences and rhythms as well. As mentioned, at present *thebeat* is focused on the auditory domain, and does not allow working with, for instance, visual stimuli or visual data. We hope to include such functionality in the future, and envision the package being used for different modalities, as well as for physiological data. Care was taken to allow for such integration in the future, and we invite anyone to contribute and suggest such functionality.

We believe that *thebeat* can serve as an open-source platform onto which the research community can build, adding in functionality that is useful for ourselves as well as for others. We also think *thebeat* constitutes a starting point for discussions about the methods that are used in rhythm and timing research (cf. Hersh et al., 2023). Since these methods are far from standardized, different methods exist for, for instance, calculating phase differences or for creating sequences that are temporally random (Madison & Merker, 2002). We hope that *thebeat* will unite researchers in finding the most reliable and accurate methods, advancing the field through open discussion.

To conclude, *thebeat* is a first step towards a more consistent way of working with temporal sequences and rhythms in the behavioral and cognitive sciences. It prevents repetitive programming, and allows its users to explore temporal data reproducibly, using simple code. We therefore expect its use to extend beyond the auditory domain to any that is concerned with temporal data.

## Declarations

### Open Practices Statement

All data and materials are available from https://osf.io/jh8ep/. All code is available via https://github.com/jellevanderwerff/thebeat.

### Supplementary materials

The supplementary materials contain code for the examples used in this article, the resulting plots and sound files, and the abridged version of the dataset from Hersh et al. (2021). It is available from https://osf.io/jh8ep/.
